# ^177^Lu-PSMA-617 and Idronoxil in Men with End-Stage Metastatic Castration-Resistant Prostate Cancer (LuPIN): Patient Outcomes and Predictors of Treatment Response in a Phase I/II Trial

**DOI:** 10.2967/jnumed.121.262552

**Published:** 2022-04

**Authors:** Sarennya Pathmanandavel, Megan Crumbaker, Andrew O. Yam, Andrew Nguyen, Christopher Rofe, Elizabeth Hovey, Craig Gedye, Edmond M. Kwan, Christine Hauser, Arun A. Azad, Peter Eu, Andrew J. Martin, Anthony M. Joshua, Louise Emmett

**Affiliations:** 1 Department of Theranostics and Nuclear Medicine, St. Vincent’s Hospital, Sydney, New South Wales, Australia;; 2 Kinghorn Cancer Centre, St. Vincent’s Hospital, Sydney, New South Wales, Australia;; 3 Garvan Institute of Medical Research, Sydney, New South Wales, Australia;; 4 St. Vincent’s Clinical School, University of New South Wales, Sydney, New South Wales, Australia;; 5 Nelune Comprehensive Cancer Centre, Prince of Wales Hospital, Sydney, New South Wales, Australia;; 6 Faculty of Medicine, University of New South Wales, Sydney, New South Wales, Australia;; 7 Department of Medical Oncology, Calvary Mater Hospital, Newcastle, New South Wales, Australia;; 8 Hunter Medical Research Institute, New Lambton Heights, Newcastle, New South Wales, Australia;; 9 Department of Medicine, School of Clinical Sciences, Monash University, Melbourne, Victoria, Australia;; 10 Department of Medical Oncology, Monash Health, Melbourne, Victoria, Australia;; 11 Peter MacCallum Cancer Centre, Melbourne, Victoria, Australia;; 12 Sir Peter MacCallum Department of Oncology, University of Melbourne, Melbourne, Victoria, Australia; and; 13 NHMRC Clinical Trials Centre, University of Sydney, Sydney, New South Wales, Australia

**Keywords:** metastatic prostate cancer, theranostics, lutetium-PSMA

## Abstract

^177^Lu-PSMA-617 is an effective therapy for metastatic castration-resistant prostate cancer (mCRPC). However, treatment resistance occurs frequently, and combination therapies may improve outcomes. We report the final safety and efficacy results of a phase I/II study combining ^177^Lu-PSMA-617 with idronoxil (NOX66), a radiosensitizer, and examine potential clinical, blood-based, and imaging biomarkers. **Methods:** Fifty-six men with progressive mCRPC previously treated with taxane chemotherapy and novel androgen signaling inhibitor (ASI) were enrolled. Patients received up to 6 doses of ^177^Lu-PSMA-617 (7.5 GBq) on day 1 in combination with a NOX66 suppository on days 1–10 of each 6-wk cycle. Cohort 1 (*n* = 8) received 400 mg of NOX66, cohort 2 (*n* = 24) received 800 mg, and cohort 3 (*n* = 24) received 1,200 mg. ^68^Ga-PSMA and ^18^F-FDG PET/CT were performed at study entry, and semiquantitative imaging analysis was undertaken. Blood samples were collected for analysis of blood-based biomarkers, including androgen receptor splice variant 7 expression. The primary outcomes were safety and tolerability; secondary outcomes included efficacy, pain scores, and xerostomia. Regression analyses were performed to explore the prognostic value of baseline clinical, blood-based, and imaging parameters. **Results:** Fifty-six of the 100 men screened were enrolled (56%), with a screening failure rate of 26% (26/100) for PET imaging criteria. All men had received prior treatment with ASI and docetaxel, and 95% (53/56) had received cabazitaxel. Ninety-six percent (54/56) of patients received at least 2 cycles of combination NOX66 and ^177^Lu-PSMA-617, and 46% (26/56) completed 6 cycles. Common adverse events were anemia, fatigue, and xerostomia. Anal irritation attributable to NOX66 occurred in 38%. Forty-eight of 56 had a reduction in prostate-specific antigen (PSA) level (86%; 95% CI, 74%–94%); 34 of 56 (61%; 95% CI, 47%–74%) had a PSA reduction of at least 50%. Median PSA progression-free survival was 7.5 mo (95% CI, 5.9–9 mo), and median overall survival was 19.7 mo (95% CI, 9.5–30 mo). A higher PSMA SUV_mean_ correlated with treatment response, whereas a higher PSMA tumor volume and prior treatment with ASI for less than 12 mo were associated with worse overall survival. **Conclusion:** NOX66 with ^177^Lu-PSMA-617 is a safe and feasible strategy in men being treated with third-line therapy and beyond for mCRPC. PSMA SUV_mean_, PSMA-avid tumor volume, and duration of treatment with ASI were independently associated with outcome.

## Visual Abstract

**Figure:**
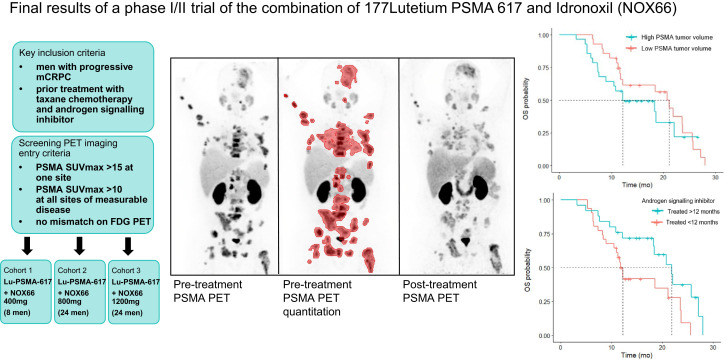

Metastatic castration-resistant prostate cancer (mCRPC) is a lethal disease, and treatment options remain limited. ^177^Lu-prostate-specific membrane antigen-617 (^177^Lu-PSMA-617) is a radioligand therapy that targets prostate-specific membrane antigen (PSMA), a receptor highly expressed on prostate cancer cells (*1*). ^177^Lu-PSMA-617 has shown promising results in prospective single-center studies, the phase II TheraP trial, and the phase III VISION trial (*2*–*5*). However, secondary treatment resistance hinders longer-term outcomes for many men (*2*,*3*,*6*).

Combination therapies may overcome resistance mechanisms and improve clinical outcomes. Idronoxil (NOX66) is a derivative of the flavonoid genistein that binds to external NADH oxidase 2, a tumor-specific enzyme that induces apoptosis and inhibits topoisomerase II. It has shown potential as a radiation sensitizer in prostate cancer (*7*–*9*). We hypothesized that combining NOX66 with ^177^Lu-PSMA-617 may improve treatment responses, with a minimal increase in toxicity.

Improving treatment response with targeted radionuclide therapy involves not only optimizing treatment responses through effective combinations but also improving patient selection. Quantitative parameters on ^68^Ga-HBEDD-PSMA-11 and ^18^F-FDG PET/CT have shown potential as predictive and prognostic biomarkers for ^177^Lu-PSMA-617 therapy (*6*,*10*–*13*). The duration of prior treatments and other markers of treatment resistance, such as androgen receptor splice variant 7, may also have prognostic utility (*11*,*14*,*15*). We report the results of a trial of combination NOX66 and ^177^Lu-PSMA-617. Additionally, we evaluate the predictive and prognostic potential of blood-based markers, clinical factors, and molecular imaging.

## MATERIALS AND METHODS

### Study Design

This was a prospective single-center phase I/II dose escalation/expansion trial of combination ^177^Lu-PSMA-617 and NOX66. The St. Vincent’s Hospital institutional review board approved the study protocol (HREC/17/SVH/19 and ACTRN12618001073291), and all patients provided written informed consent.

### Screening

Men with mCRPC experiencing progression on conventional imaging (CT and bone scanning) or a rising level of prostate-specific antigen (PSA) based on Prostate Cancer Working group 3 criteria (*16*), and previously treated with at least 1 line of taxane chemotherapy (docetaxel or cabazitaxel) and at least 1 androgen signaling inhibitor (ASI) (abiraterone or enzalutamide), were screened. All patients had adequate organ function (baseline hemoglobin ≥ 100 g/L, platelet count ≥ 100 × 10^9^/L, and estimated glomerular filtration rate ≥ 40 mL/min), an estimated life expectancy of more than 12 wk, and a World Health Organization Eastern Cooperative Oncology Group performance status of no more than 2.

**FIGURE 1.**
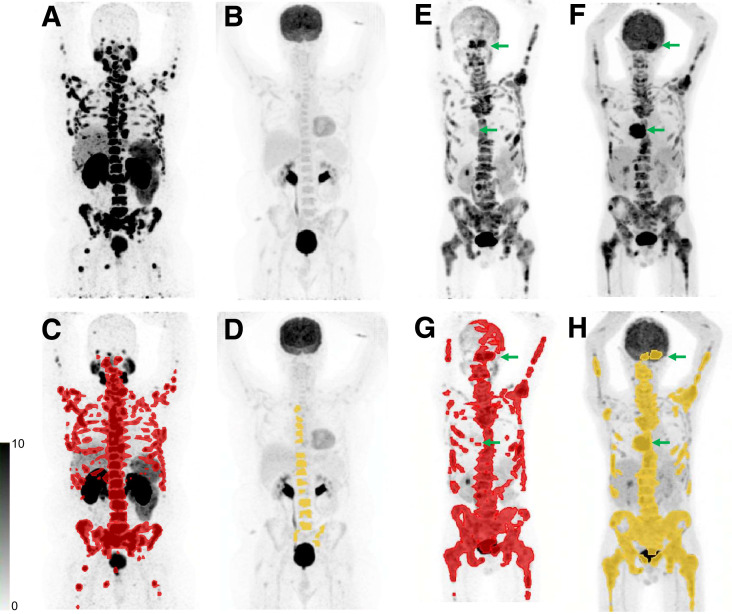
(A–D) Patient who was eligible on the basis of imaging with PSMA-avid disease (A) and no sites of discordant ^18^F-FDG (B). Quantitative PSMA tumor volume (C) and ^18^F-FDG tumor volume (D) are also shown. (E–H) Patient who was ineligible on the basis of imaging showing 2 sites (arrows) with higher ^18^F-FDG avidity (F) than PSMA avidity (E). Quantitative PSMA tumor volume (G) and ^18^F-FDG tumor volume (H) are also shown.

Men underwent screening with ^18^F-FDG and PSMA PET/CT, bone scanning, and CT and were eligible if they had an SUV_max_ of more than 15 on PSMA PET at 1 or more sites, an SUV_max_ of more than 10 at all measurable sites, and no ^18^F-FDG avidity without corresponding PSMA uptake (Fig. 1).

### Study Treatment

All men received up to 6 cycles of ^177^Lu-PSMA-617 at 6-wk intervals in combination with 1 of 3 doses of NOX66 (400, 800, and 1,200 mg). NOX66 was administered via suppository on days 1–10 after each ^177^Lu-PSMA-617 injection. All cohorts were administered 7.5 GBq of ^177^Lu-PSMA-617 on day 1 via a slow intravenous injection. In addition, participants in cohort 1 (*n* = 8) received 400 mg of NOX66. After interim safety data reviews, the dose of NOX66 was escalated to 800 mg for cohort 2 (*n* = 24) and 1,200 mg for cohort 3 (*n* = 24).

The PSMA-617 precursor (AAA, Novartis) was radiolabeled to no-carrier-added ^177^Lu-chloride according to the manufacturer’s instructions by a qualified radiopharmacist or radiochemist. Quality control tests for radionuclidic and radiochemical purity were performed using high-pressure liquid chromatography and thin-layer chromatography. NOX66 (Noxopharm Ltd.) was commercially produced.

###  Imaging Procedures and Analysis

^68^Ga-HBEDD-CC PSMA-11 was produced on-site in compliance with good laboratory practices using a Trasis automated radiopharmacy cassette. ^18^F-FDG was produced off-site commercially. Radiopharmacy quality control testing used a high-pressure liquid chromatography method. Patients were injected with a 2.0 MBq/kg dose of PSMA and a 3.5 MBq/kg dose of ^18^F-FDG, with identical imaging parameters (dose, time after injection, and imaging protocols) for each patient. All PET/CT imaging was undertaken using a Phillips Ingenuity TOF-PET/64-slice CT scanner. An unenhanced low-dose CT scan was performed 60 min after tracer injection. Immediately after CT, a whole-body PET scan was acquired for 2 min per bed position.

PET/CT scans were analyzed semiquantitatively using MIM software and a standardized semiautomated workflow to delineate regions of interest with a minimum SUV_max_ cutoff of 3 for PSMA and blood pool intensity, plus 1.5 SDs for ^18^F-FDG (*17*). Quantitation derived total metabolic tumor volume, SUV_max_, SUV_mean_, and total lesional activity for both ^18^F-FDG and PSMA (MIM Software).

### Study Endpoints

Safety and tolerability were assessed using the National Cancer Institute Common Terminology Criteria for Adverse Events (version 5.0) every 2 wk during each 6-wk cycle until 6 wk after the final study treatment. To assess efficacy, we measured the PSA decline from baseline (absolute and ≥50% [PSA50]) at any time point, PSA progression-free survival (PFS), and overall survival (OS). Time-to-event endpoints (PSA PFS and OS) were defined as the interval from the date of enrollment to the event date, or the date last known to be event-free (at which point the observation was censored). Patient-reported outcomes were measured on day 1 of each cycle and during follow-up using the University of Michigan xerostomia-related quality-of-life scale (XeQoLS) (*18*) and the short form of the brief pain inventory (*19*). Pain palliation was defined as a reduction by at least 30% in the worst pain intensity score over the last 24 h observed at 2 consecutive evaluations (*20*).

### Clinical, Blood-Based, and Molecular Analysis

Clinical information regarding initial diagnosis, Gleason score, previous lines of therapy, and prior treatment responses was collected. Blood was prospectively collected for biomarker measurement, including hemoglobin, platelets, alkaline phosphatase, albumin, and PSA. Whole-blood samples were collected at baseline, before cycle 3, and before cycle 6 for analysis of potential molecular biomarkers, including androgen receptor splice variant 7. Androgen receptor splice variant 7 analysis was performed using the method described by To et al. (*21*).

### Statistical Analyses

The study sample size was calculated to characterize the toxicity profile of the combination, based on the expectation that an adverse event with a true 5% incidence would be detected with 70% probability in a sample of 24 and detected with over 90% probability in a sample of 56. All patients who received at least 1 cycle of study treatment were included in the safety and efficacy analyses. *P* values below 5% were considered significant but interpreted cautiously. A 2-sided exact binomial 95% CI was calculated for PSA response rates. The Kaplan–Meier method was used to characterize time-to-event endpoints (PSA PFS, and OS) and to estimate medians (presented with 95% CIs). The 3 NOX66 dose levels were compared in terms of adverse events, PSA50, and OS.

Cox proportional-hazards regression, and logistic regression, were used to identify prognostic factors for time-to-event (PSA PFS, and OS) and binary endpoints (PSA50), respectively. The covariates investigated included baseline clinical, blood-based, and imaging parameters, including tumor volume and intensity scores (SUV_max_ and SUV_mean_). In the absence of compelling evidence of a dose–response effect on PSA50, the cohorts were grouped, and prognostic analyses were performed on the grouped cohort.

We used the relaxed LASSO (least absolute shrinkage and selection operator) regression method to identify covariates for inclusion in a multivariable model (*22*). These were fitted in a standard multivariable Cox regression model to obtain conventional hazard ratios (HRs), 95% CIs, and *P* values.

All patients with a worst pain score of at least 4 were included in the analysis, and changes in score between baseline, precycle 3, and end of treatment were compared. Scores from the XeQoLS questionnaire were compared between baseline, precycle 3, and end of treatment. A 2-tailed paired *t* test was used to assess for a change in scores. Analyses were performed using R (version 4.0.5) and SPSS (version 25).

## RESULTS

### Baseline Patient Characteristics

One hundred men were screened, of whom 56 (56%) were enrolled between November 2017 and February 2020. Twenty-six percent were ineligible on the basis of the PET imaging criteria (13% because of low-PSMA-intensity disease and 13% because of sites with ^18^F-FDG/PSMA mismatch). Remaining screening fails (18%) were from clinical deterioration (6%), concurrent illness (3%), low hemoglobin (7%), or personal reasons (2%). Baseline characteristics are summarized in Table 1. All patients had prior treatment with at least 1 ASI and taxane chemotherapy, with 95% (53/56) having 2 lines of taxane chemotherapy; 66% (37/56) had at least 20 PSMA-avid metastases, 88% (49/56) had metastases in bone, 55% (31/56) had metastases in lymph nodes, and 19% (11/56) had visceral metastases.

**TABLE 1:** Patient Characteristics (*n* = 56)

Characteristic	Data
Age (y)	69 (64–74)
Eastern Cooperative Oncology Group status	
0 or 1	49 (88)
2	7 (12)
PSA at C1 (μg/L)	115 (46–476)
Hemoglobin (reference range [RR], 130–180 g/L)	122 (110–131)
Alkaline phosphatase (RR, 30–100 U/L)	113 (86–231)
Albumin (RR, 36–52 g/L)	38 (34–41)
De novo metastatic disease	29 (52)
Gleason score	
≤7	9 (16)
8–10	35 (63)
Unknown/not available	12 (21)
Prior systemic treatments	
Luteinizing hormone–releasing hormone agonist or antagonist	56 (100)
Chemotherapy	56 (100)
Docetaxel	56 (100)
Cabazitaxel	53 (91)
Other chemotherapy	5 (9)
ASI	56 (100)
Enzalutamide only	27 (48)
Abiraterone only	13 (23)
Abiraterone + enzalutamide	16 (29)
Clinical trial medication	4 (7)
PSMA PET	
SUV_mean_	8 (7–10)
SUV_max_	39 (29–61)
Volume (L)	0.64 (0.19–1.21)
^18^F-FDG PET	
SUV_mean_	4 (3–5)
SUV_max_	8 (5–10)
Volume (L)	0.07 (0.02–0.31)
Disease volume	
<20 metastases	19 (33)
≥ 0 metastases	37 (66)
Sites of disease on PSMA PET	
Bone	49 (88)
Lymph node	31 (55)
Viscera	12 (21)

Qualitative data are absolute counts and percentage; continuous data are median and interquartile range.

Because of the small numbers in each NOX66 dose cohort, with ^177^Lu-PSMA-617 as the key treatment, we combined the 3 patient cohorts for reporting of outcomes and for exploratory analysis of biomarkers of response and survival. Analysis of the 3 dose escalation cohorts of NOX66 did not reveal any statistical differences in adverse events, PSA response rate, PSA PFS, or OS.

### Safety and Tolerability

Adverse events were predominantly grade 1 (149/188; 79%). The most common toxicities were anemia (50/56; 89%), fatigue (36/56; 64%), and xerostomia (33/56; 59%) (Table 2). Anal inflammation due to the NOX66 suppository occurred in 38% (21/56), with 27% (15/56) requiring topical treatment for anal inflammation. The rate of grade 1 anal inflammation was higher in cohort 3 (46%) than in cohort 1 or 2 (25% and 21%, respectively). Two men in cohort 2 and 1 man in cohort 3 required dose reduction or omission of NOX66. Four cases of grade 3 anemia were reported. There were no other significant differences in toxicities across the 3 cohorts and no grade 4–5 adverse events or treatment-related deaths.

**TABLE 2:** Summary of Common and Therapeutically Relevant Adverse Events (*n* = 56)

Adverse event	Grade 1	Grade 2	Grade 3	All grades
Anemia	31 (55)	16 (29)	4 (7)	51 (91)
Xerostomia	30 (54)	3 (5)	0 (0)	33 (59)
Fatigue	27 (48)	8 (14)	0 (0)	35 (63)
Anal inflammation	18 (32)	3 (5)	0 (0)	21 (38)
Nausea	15 (27)	0 (0)	0 (0)	15 (27)
Thrombocytopenia	12 (21)	3 (5)	0 (0)	15 (27)
Constipation	11 (20)	1 (2)	0 (0)	12 (21)
Neutropenia	5 (9)	0 (0)	0 (0)	5 (9)
Pneumonitis*	0 (0)	1 (3)	0 (0)	1 (3)

*Attributed to radiation therapy prior to enrollment. Data are number followed by percentage.

### Treatment Duration

Participants received a median of 5 (interquartile range, 3–6) cycles of ^177^Lu-PSMA-617 and NOX66; 96% (54/56) received at least 2 cycles, and 46% (26/56) completed all 6 cycles. Of the 30 participants who ceased treatment before completing 6 cycles, 2 participants ceased because of exceptional responses, and the other patients ceased because of progressive disease (46%, *n* = 26), withdrawal of consent (2%, *n* = 1), or inability to continue the study because of COVID-19 travel restrictions (2%, *n* = 1). One participant ceased NOX66 because of grade 2 anal inflammation but continued ^177^Lu-PSMA-617. No participants ceased LuPSMA-617 because of toxicity.

### Treatment Response

**FIGURE 2.**
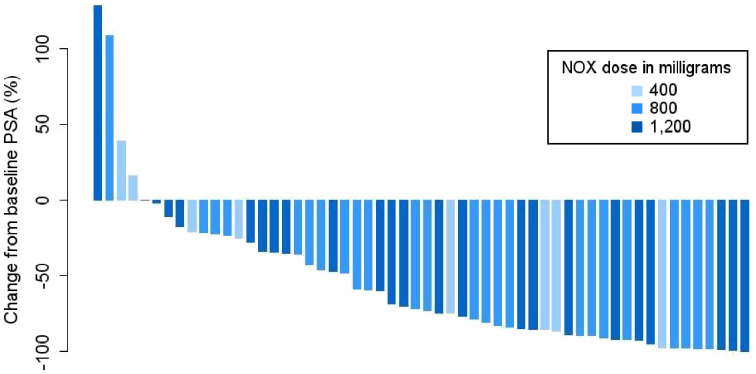
Waterfall plot of best PSA responses as indicated by maximum percentage change from baseline at any time point. NOX = NOX66.

**FIGURE 3.**
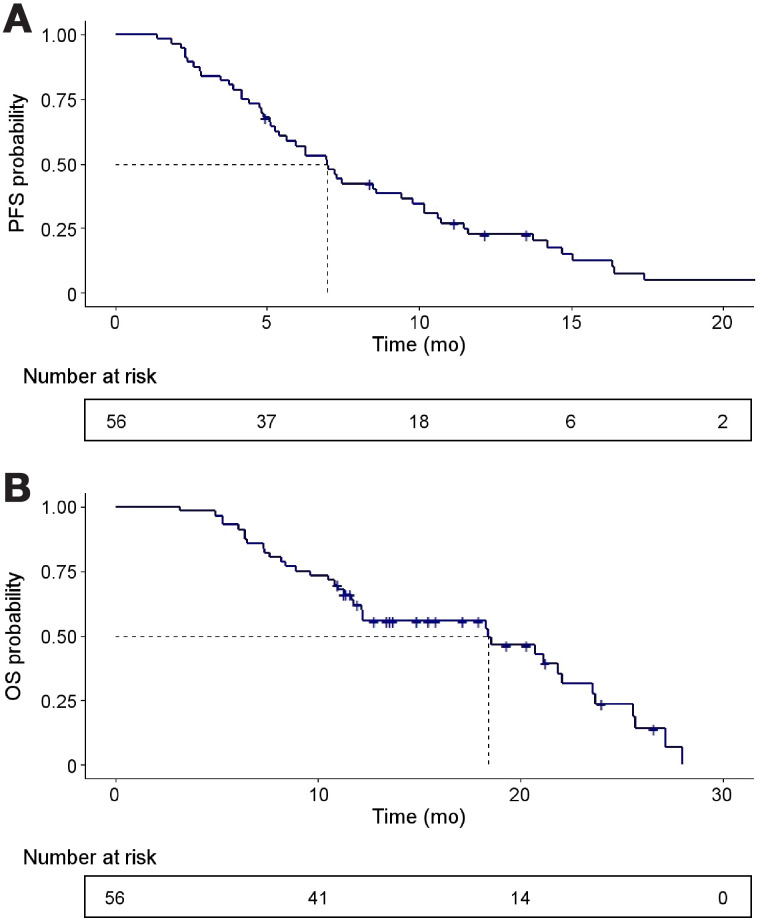
Kaplan–Meier curves for PSA progression-free survival (A) and OS (B).

At a median follow-up of 21.8 mo, PSA50 occurred in 61% (34/56; 95% CI, 47%–74%), whereas any decline in PSA occurred in 86% (48/56; 95% CI, 74%–94%). The waterfall plot of best PSA responses at any time point is shown in Figure 2. At the time of this analysis, 91% (51/56) of participants have had PSA progression and 66% (37/56) have died. The median PSA PFS was 7.5 mo (95% CI, 5.9–9.0 mo) (Fig. 3A), and the median OS was 19.7 mo (95% CI, 9.5–30.0 mo) (Fig. 3B).

### Quality of Life

The baseline brief pain inventory assessment (short form) was completed by 95% (53/56) of the men. The mean worst pain score at baseline was 4.21 (range, 0–10; SD, 2.99). Fifty-six percent (29/52) of the men recorded a worst pain score of at least 4, and of these, 41% (12/29) experienced pain palliation at any time point.

The baseline XeQoLS assessment was completed by 48 (86%) of 56 men at baseline, with serial results at cycle 3 (48/56) and cycle 6 (26/48). There was no significant difference in XeQoLS scores between baseline and cycle 3, but a statistically significant difference was found between baseline and cycle 6 (*P* = 0.04). There were no differences in XeQoLS scores among the 3 dose levels.

### Potential Prognostic Factors

We performed exploratory univariable analysis to identify potential markers of PSA50 and OS.

#### Quantitative PET Imaging Markers

Comparative screening imaging characteristics are detailed in Table 1. A higher tumor volume on PSMA PET was associated with a lower likelihood of achieving PSA50 (odds ratio [OR], 0.41 [95% CI, 0.19–0.87]; *P* = 0.02) and shorter OS (HR, 2.18 [95% CI, 1.36–3.51]; *P* = 0.001). PSMA SUV_mean_ was associated with an increased likelihood of achieving PSA50 (OR, 1.57 [95% CI, 1.12–2.19]; *P* = 0.008). A higher ^18^F-FDG–avid tumor volume at baseline was associated with a worse OS (HR, 3.02 [95% CI, 1.04–8.79]; *P* = 0.04). The presence of visceral metastases was also associated with a worse OS (HR, 2.35 [95% CI, 1.06–5.20]; *P* = 0.04).

#### Impact of Prior Treatments on Outcome

The most common treatment immediately before enrollment in the trial was cabazitaxel (70%, 39/56). Receiving either chemotherapy or ASI immediately before the trial did not predict treatment response or survival. Similarly, the duration of chemotherapy did not predict a response to therapy. However, an ASI treatment duration of more than 12 mo was significantly associated with improved OS (HR, 0.45 [95% CI, 0.22–0.91]; *P* = 0.03).

#### Blood-Based Markers

A higher baseline level of hemoglobin was associated with higher odds of PSA50 (OR, 1.05 [95% CI, 1.01–1.10]; *P* = 0.03) and improved OS (HR, 0.96 [95% CI, 0.93–0.99]; *P* = 0.004). Other known prognostic markers, including baseline alkaline phosphatase, PSA, and use of opioid analgesia, did not correlate with outcome.

Thirty-five patients had androgen receptor splice variant 7 (ARV7) assessed before cycle 1; of these, 9 (26%) were ARV7-positive. Two patients remained positive at cycle 3, and 2 patients became positive while on treatment. A total of 11 patients had ARV7 detected at any time point. The presence of ARV7 at any time point was not significantly associated with treatment response or survival.

#### Multivariable Analysis of Potential Prognostic Factors

**FIGURE 4:**
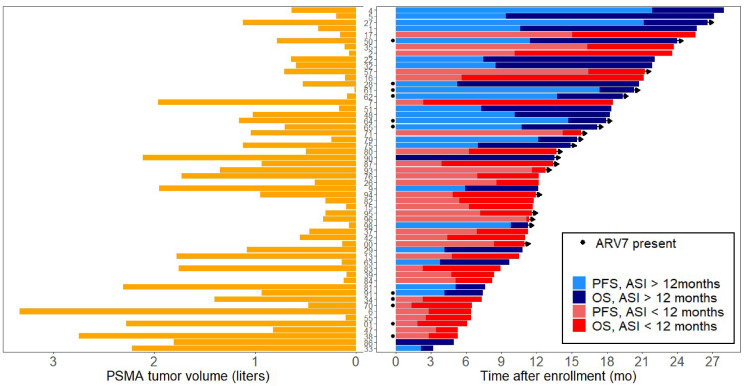
Graphical representation of important markers of OS. (Right) Swimmer plot showing individual-patient PFS and OS. (Left) Graph showing corresponding baseline tumor volume for each patient.

A higher PSMA mean intensity score (SUV_mean_) (OR, 1.61 [95% CI, 1.12–3.32]; *P* = 0.01) and a lower PSMA tumor volume (OR, 0.42 [95% CI, 0.18–0.94]; *P* = 0.04) remained predictive of PSA50, whereas PSMA tumor volume (HR, 2.19 [95% CI, 1.38–3.46]; *P* = 0.001) predicted a worse OS (Fig. 4). The only clinical parameter predictive of survival was treatment with ASI for more than 12 mo (HR, 0.42 [95% CI, 0.20–0.87]; *P* = 0.02). Baseline ^18^F-FDG tumor volume, presence of visceral disease, and hemoglobin did not remain independently predictive of outcome (Tables 3 and 4).

**TABLE 3:** Final Multivariable Model for Association of Baseline Markers with PSA50

	LASSO OR	Multivariable logistic regression			Backward elimination model		
Variable		OR	95% CI	*P*	OR	95% CI	*P*
PSMA TV	0.73	0.47	0.20–1.09	0.08	0.42	0.18–0.94	0.04
PSMA SUV_mean_	1.20	1.61	1.10–2.34	0.01	1.61	1.12–2.32	
Hemoglobin	1.02	1.04	0.99–1.10	0.12	NA	NA	NA

TV = tumor volume; NA = not applicable.

**TABLE 4:** Final Multivariable Model for Association of Baseline Markers with OS

	LASSO OR	Multivariable Cox regression			Backward elimination model		
Variable		HR	95% CI	*P*	HR	95% CI	*P*
PSMA TV	1.67	2.05	1.19–3.53	0.009	2.19	1.381–3.463	0.001
ASI > 12 mo	0.70	0.56	0.24–1.31	0.56	0.42	0.202–0.869	0.02
^18^F-FDG tumor volume (L)	NA	0.99	0.25–3.98	0.99	NA	NA	NA
Hemoglobin	0.99	0.98	0.95–1.02	0.30	NA	NA	NA
Presence of visceral disease	1.499	2.01	0.89–4.53	0.09	NA	NA	NA

TV = tumor volume; NA = not applicable.

## DISCUSSION

PSMA-targeted radionuclide therapy is emerging as a new treatment paradigm in men with mCRPC. The randomized TheraP trial demonstrated a significantly improved treatment response (PSA50), PFS, and quality-of-life parameters for ^177^Lu-PSMA-617, compared with cabazitaxel chemotherapy, in mCRPC. However, whereas results from TheraP are encouraging, PFS remains short, with a median of 5.1 mo (95% CI, 3.4–5.7) (*4*). We postulate that deepening and prolonging responses to ^177^Lu-PSMA-617 therapy for men with mCRPC may be possible by targeting intracellular resistance mechanisms to maximize treatment effect. This article reports the results of a dose escalation trial of ^177^Lu-PSMA-617 with a radiation sensitizer (NOX66) and evaluates potential predictive markers of response to PSMA-targeted therapy.

Treatment response rates to the ^177^Lu-PSMA-617 and NOX66 combination were high with a 61% PSA50, even though most men in this trial had high-volume disease, baseline anemia, high baseline opiate requirements, and 95% had undergone 2 lines of taxane therapy. Despite these high-risk features, the treatment response rate is in line with previous prospective single-center trials and the TheraP study (range, 36%–66%) (*2*–*4*). Further, PFS and OS were longer than those reported in previous studies with ^177^Lu-PSMA-617 and longer than those reported using alternative treatments after taxane chemotherapy (*3*,*23*). These results are encouraging for men with ASI and taxane-refractory mCRPC, but a randomized trial—possibly with less stringent imaging enrollment criteria—will be required to determine whether these results are due to the novel treatment combination or to patient selection.

NOX66 was included in the trial as a potential tumor-specific radiation sensitizer that binds to external NADH oxidase 2, a tumor-specific enzyme inducing apoptosis and inhibiting topoisomerase II and demonstrating radiation sensitization in preclinical models (*7*). We did not find an association between an increasing dose of NOX66 and PSA50 or OS. However, the role of NOX66 in the study was as a radiation sensitizer, rather than having a direct effect on therapy, and it may be that either the lower dose of NOX66 was sufficient to induce radiation sensitivity or the impact of NOX66 was limited. The safety of combination therapy with ^177^Lu-PSMA-617 and NOX66 has been previously reported for the first 2 cohorts of the LuPIN trial and was confirmed in this study (*24*).

Predictors of treatment response are important to further improve PSMA-targeted therapy. Men were screened for this study with molecular imaging, with a requirement for an SUV_max_ of at least 15 on PSMA PET and no sites of ^18^F-FDG/PSMA PET mismatch. An SUV_max_ of at least 15 has been previously reported to stratify men into responders and nonresponders for ^177^Lu-PSMA-617 therapy (*2*). Hence it is not surprising that PSMA SUV_max_ was not predictive of a treatment response in this study. However, PSMA SUV_mean_ was an independent predictor of treatment response in this study and previously (*10*). A relationship between a higher SUV_mean_ and improved clinical outcomes is biologically plausible. Intra- and interlesional heterogeneity of PSMA is common in mCRPC, and high heterogeneity of expression is likely to impact treatment response (*25*). SUV_mean_ is likely a better indicator of lesional heterogeneity than is SUV_max_. Further, dosimetry studies have shown that SUV_mean_ correlates with the mean absorbed radiation dose and treatment response (*13*). Although SUV_mean_ requires quantitative analysis, its repeated association with treatment response suggests that it may have a future role as a predictive biomarker for PSMA-targeted radionuclide therapy.

PSMA tumor volume at baseline was the strongest independent predictor of treatment response and was also prognostic for OS. ^18^F-FDG tumor volume was also prognostic, but not independently of other variables. Essentially, men with higher tumor volumes responded poorly to treatment. This finding agrees with retrospective analyses of men undergoing ^177^Lu-PSMA-617 therapy (*12*,*26*) and raises questions about the timing of PSMA-targeted therapy in men with mCRPC, suggesting that earlier referral for treatment after prior treatment failure may both improve treatment responses and prolong survival.

The duration of response to prior therapies may help predict the treatment response to PSMA-targeted agents and OS. We found that men with a shorter duration of response to ASI (<12 mo) had a worse OS, though the depth of the treatment response was not affected. By contrast, the duration of the response to chemotherapy, or whether the patient received chemotherapy or ASI immediately before the trial, was not predictive of either survival or response.

This study enrolled a population of heavily pretreated men with mCRPC; therefore, the identified prognostic markers may not be generalizable to other stages of prostate cancer. Larger studies are needed to validate the prognostic markers identified in this study.

## CONCLUSION

^177^Lu-PSMA-617 in combination with NOX66 is a safe treatment for heavily pretreated men with mCRPC, with encouraging results that warrant further evaluation. PSMA SUV_mean_ and tumor volume merit further investigation as imaging markers of treatment response and survival.

## DISCLOSURE

This investigator-initiated study was sponsored by St. Vincent’s Hospital Sydney and supported by a Cancer Institute NSW prostate translational research grant. Noxopharm Limited provided funding for drug and PET scans, and AAA/Novartis provided PSMA-617 ligand. Edmond Kwan receives honoraria from Janssen, Ipsen, and Astellas Pharma; has a research review, consulting, or advisory role with Astellas Pharma, Janssen and Ipsen; and receives research funding from Astellas Pharma and AstraZeneca. Anthony Joshua has an advisory role with Noxopharm Limited and receives institutional funding from Novartis. Louise Emmett has an advisory role with Noxopharm Limited and receives trial support from Novartis and Astellas. No other potential conflict of interest relevant to this article was reported.

## Notes

KEY POINTS **QUESTION:** Is combination therapy with ^177^Lu-PSMA-617 and NOX66 feasible and safe? **PERTINENT FINDINGS:** This phase I/II dose escalation and expansion study found that the combination is feasible and potentially efficacious. Evaluation of clinical, blood-based, and quantitative imaging markers identified PSMA SUV_mean_, tumor volume, and duration of prior treatment with ASI as potential prognostic markers. **IMPLICATIONS FOR PATIENT CARE:** Further randomized studies combining ^177^Lu-PSMA-617 and NOX66 are needed. Quantitative imaging markers correlate with treatment response and survival and should be explored further.
